# Framework for the impact analysis and implementation of Clinical Prediction Rules (CPRs)

**DOI:** 10.1186/1472-6947-11-62

**Published:** 2011-10-14

**Authors:** Emma Wallace, Susan M Smith, Rafael Perera-Salazar, Paul Vaucher, Colin McCowan, Gary Collins, Jan Verbakel, Monica Lakhanpaul, Tom Fahey

**Affiliations:** 1 Department of General Practice, Royal College of Surgeons in Ireland, (123 Stephen's green) Dublin 2, Republic of Ireland; 2 Department of Primary Health Care, University of Oxford, (23-38 Hythe Bridge Street), Oxford, (OX1 2ET), UK; 3 Department of Community Medicine and Primary Care, University of Geneva, (Michel-Servet 1, CH-1211), Geneva, Switzerland; 4 School of Medicine, University of Dundee, (Nethergate), Dundee, (DD1 4HN), UK; 5 Centre for Statistics in Medicine, University of Oxford, (Linton Road), Oxford, (OX2 6UD), UK; 6 Department of General Practice, Katholieke University, Leuven, Belgium; 7 Department of Medical and Social Care Education, University of Leicester, (University road), Leicester, (LE1 7RH), UK

## Abstract

Clinical Prediction Rules (CPRs) are tools that quantify the contribution of symptoms, clinical signs and available diagnostic tests, and in doing so stratify patients according to the probability of having a target outcome or need for a specified treatment. Most focus on the derivation stage with only a minority progressing to validation and very few undergoing impact analysis. Impact analysis studies remain the most efficient way of assessing whether incorporating CPRs into a decision making process improves patient care. However there is a lack of clear methodology for the design of high quality impact analysis studies.

We have developed a sequential four-phased framework based on the literature and the collective experience of our international working group to help researchers identify and overcome the specific challenges in designing and conducting an impact analysis of a CPR.

There is a need to shift emphasis from deriving new CPRs to validating and implementing existing CPRs. The proposed framework provides a structured approach to this topical and complex area of research.

## Background

The International Diagnosis and Prognosis Prediction (IDAPP) group has recently been established. This collaborative group includes researchers and clinicians with an interest in Clinical Prediction Rules (CPRs). One of its objectives is to enhance the analysis and reporting of CPR research. One area of interest is the impact analysis of CPRs. An obstacle to this type of research is the lack of clear and well-disseminated methodology for the design of high quality impact studies. At a recent IDAPP workshop a sequential four-phased framework was developed to help researchers identify and overcome the specific challenges in designing and conducting an impact analysis of a CPR. This paper presents an overview of this framework.

**Figure 1:**
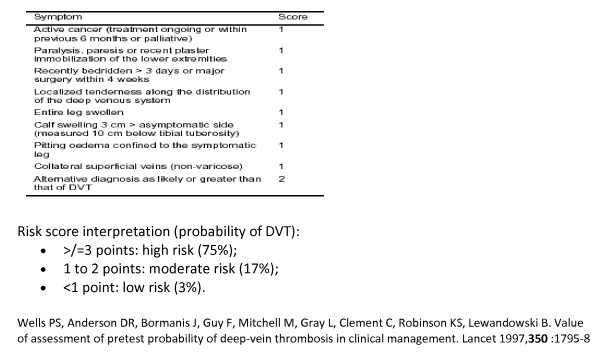
**Alternative formats and functions of Clinical Prediction Rules (CPRs)**. Assistive CPR; Wells CPR for Deep Venous Thrombosis (DVT)

**Figure 2:**
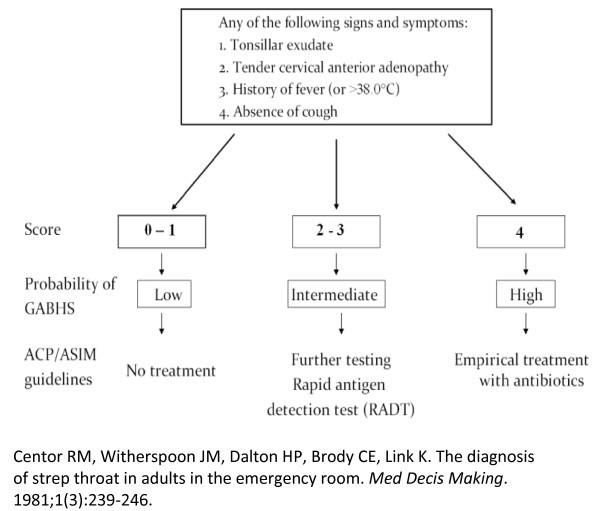
**Alternative formats and functions of Clinical Prediction Rules (CPRs)**. Directive CPR; Centor score for sore throat

A CPR has been defined as a tool that uses a combination of history, clinical examination and diagnostic tests to stratify a patient in terms of the probability of having a target outcome [1]. CPRs may relate to diagnosis, prognosis or treatment and include scoring systems which predict outcomes or inform management decisions, risk calculators and may also encompass screening questionnaires. There are an increasing number of CPRs included in clinical guidelines and implemented in clinical management systems such as GP software [2]. CPRs may be assistive and therefore designed to calculate probabilities without recommending decisions or directive and designed to give specific management recommendations (Figure 1 and Figure 2).

**Figure 3:**
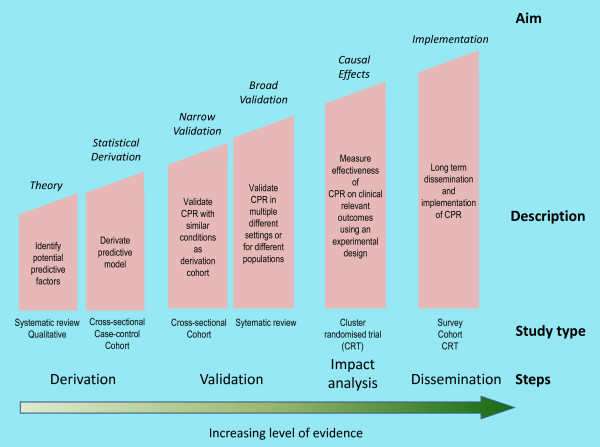
**Theoretical framework for study designs from theory to implementation of CPRs**.

There is a widely accepted methodology for the development of CPRs [1,3]. The ***derivation ***of a CPR is the first of three steps required before it can be disseminated and used in practice. This is followed by internal and external ***validation ***(Step Two) before finally testing the ***impact ***(Step Three) of its use on clinical outcomes. These steps require cumulative levels of evidence and the adoption of several types of study designs to answer the relevant research and clinical questions (Figure 3).

The increasing number of CPRs reported in the literature have a tendency to focus on the derivation stage with only a minority progressing to validation and very few undergoing impact analysis [4]. Nevertheless, impact analysis studies remain the most valid way of assessing whether incorporating CPRs into a decision making process improves patient outcomes. There is a need to change emphasis from deriving new CPRs to validating and implementing existing CPRs.

**Figure 4:**
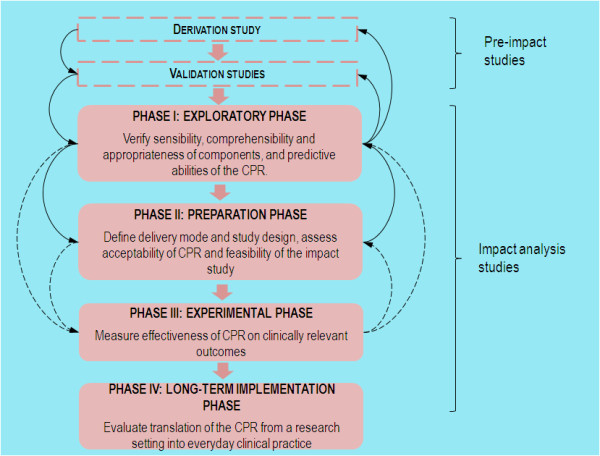
**Phases for impact analysis of CPRs**.

The integration of a validated CPR into routine clinical practice presents a number of challenges. These include measuring the acceptability of the CPR to clinicians, deciding how it will be delivered at the point of care and the applicability of a CPR derived in one setting to a new setting. As a result, we have developed a tailored four-phased framework based on the literature and the collective experience of our working group (Figure 4) [4-6]. Although the phases in the framework are designed to be sequential, there may be a requirement to adopt an iterative process where findings from a later phase may prompt reassessment of earlier work.

### Phase I: Exploratory phase: Is this CPR ready for impact analysis?

For a CPR to be ready for impact analysis it needs to have been derived and broadly validated using pre-defined methodological criteria [1]. This should ensure the components of the CPR (a combination of symptoms, signs and diagnostic tests) are clinically sensible, comprehensive and appropriate for the purpose of the rule. The most appropriate study design for derivation and validation will depend on the purpose of the rule (i.e. assistive or directive). Considerations of prognostic and diagnostic variables are best addressed utilising a cohort or cross sectional study design. Variables concerned with treatment effect modifiers will necessitate consideration of an alternative study design [7]. A systematic review is the best method for identifying and analysing all validation studies available for the target CPR. At the end of phase I the CPR should be finalised and if any adjustments were to occur to the CPR, effects on validity should be re-assessed. The absence of such a validation would need to be justified with further analysis incorporated into ongoing work. For example, in a large impact analysis study focusing on the Goldman CPR, used in the triage of patients in the emergency department with suspected acute cardiac ischaemia, clinicians sought to increase the sensitivity of the rule by adding the presence of an electrocardiogram predictor variable to the existing rule [8]. This remodeling reflected the reality of clinical practice and ensured greater clinician confidence in using the rule. In this instance, clinicians agreed to provide data needed to measure the original rule and the modified version to allow analysis of the impact of these modifications.

### Phase II: Preparation phase

The aim of this phase is to set the groundwork for the impact analysis in a defined setting. This includes assessing acceptability and identifying potential barriers to the use of the CPR. For instance, an Australian impact analysis study of the Ottawa ankle rule considered and addressed barriers at an organisational, individual and societal level before conducting their study [9]. From an organisational perspective, clinical preparation is essential and a useful strategy to engage clinicians is the use of a simulation exercise prior to study implementation. The impact analysis study of the Goldman CPR for patients with suspected cardiac ischaemia presenting to A+E used this strategy with success [8].

Another important consideration is determining how the CPR will be integrated into the clinical workflow. This may be achieved in different ways, for instance, incorporation of the CPR as part of a broader guideline implementation and embedding the CPR into clinical software or a computerised clinical decision support system (CDSS) [10,11]. Consideration may be given to providing feedback to clinicians during the trial as this has been shown to be an effective means of maintaining participant interest in the implementation trial of the Ottawa ankle rule [12].

Successful development of the CPR should lead to the formulation of an intervention that can be pilot tested and is ready to proceed to the next phase.

### Phase III: Experimental phase, impact analysis

The aim of this phase is to determine whether the CPR is effective-does it improve the process of clinical care, improve patient outcomes and/or increase cost-effectiveness [1]. Whilst impact studies using the optimal cluster RCT design are rare there are good examples [11,13,14]. An alternative design is the controlled before-and-after study, where study outcomes are measured before, during, and after using the CPR and compared to the same outcomes with a control group in which the CPR was not used [8,12]. On-off or interrupted time series study designs may also be used but are subject to bias due to temporal trends [10].

Potential sources of bias during impact analysis are similar to those for randomised controlled trials (RCTs). In terms of study design there should be a control/comparison arm and a cluster design will ensure minimal contamination of control group patients. There should be at least one intervention arm but consideration may be given to having a number of trial arms with varying levels of support surrounding the CPR. For instance, a pneumonia guideline implementation study which included the Pneumonia Severity Index CPR used low, moderate and high intensity guideline implementation strategies [15]. A mixed methods approach may be useful where a qualitative evaluation is nested within the RCT design in order to assess the uptake, feasiblility and clinicians/patients attitudes and experiences in relation to the specific CPR implementation [16].

Evaluating the impact of a CPR requires careful consideration of suitable outcome measures. The type of outcome should be clearly identified and may relate to process of care, physician behavior, patient outcomes or multiple endpoints. The choice of outcome measure(s) will be influenced by whether the CPR being tested is assistive or directive in nature [4]. Blinding of outcome assessment is particularly important in the context of CPRs as knowledge of the outcome may influence how the CPR is scored. An interesting way to address this may be to embed the CPR into clinical software to facilitate blinding of the underlying CPR implementation details. For example, a study examining the Acute Cardiac Ischaemia Time-insensitive Predictive Instrument (ACI-TIPI) embedded the CPR in the electrocardiogram report. In this on-off study, during intervention periods the probability of cardiac ischaemia was automatically printed on the ECG while during control periods only the standard header text was printed [10].

Appropriate sample size calculation will depend on the choice of the primary outcome(s) to evaluate the effectiveness of the CPR. There is an inherent trade-off in the choice of primary end-point and the number of participating centres and individuals required that experience the targeted outcome. Follow-up should involve an iterative process to identify strengths and weaknesses of the CPR and assessment of the primary outcomes in terms of CPR impact. Clinician attitudes on acceptibility regarding use of CPR may be formally evaluated with the development of a tool such as the validated Ottawa Acceptibility of Decision Rule instrument (OADRI) [17]. This simple twelve item instrument was developed to measure the acceptability of the Canadian head CT rule and the Canadian C-spine rule CPRs amongst users. It includes questions pertaining to CPR use, consistency of use and the effect geographical location has on CPR implementation. Similar context specific tools have much potential in terms of gauging opinions on CPR use and perceived barriers to utilisation.

Other important considerations are patient satisfaction and quality of life measures and these have been incorporated into study design in some implementation studies. An example is the use of the Short-Form 36 Physical Component summary scale to assess quality of life measures in a large controlled trial of a critical pathway for pneumonia which included the Pneumonia Severity Index CPR. This score was the primary outcome for this trial [18].

Important lessons can be learned from failed impact analysis CPR studies. Was the failed implementation related to the CPR or was it the implementation system? The Canadian CT Head rule study failed to reduce imaging rates in participating A+ E departments despite the investigators having much experience in this type of study design and previously successfully implementing the Ottawa ankle and knee rules and the Canadian C-Spine CPR [19]. The authors suggest that physicians tend to be risk adverse and therefore the more serious the suspected underlying clinical condition, the more likely the clinician is to order imaging regardless of CPR findings. This is very understandable considering the consequences of failing to diagnose a serious condition. Another study which incorporated the New Zealand cardiovascular risk score into a CDSS did not confer any benefit compared with chart guidelines in relation to management of hypertension in primary care [11]. The authors highlighted the restricted ability of the software programme used in this study as a potential reason for this.

These examples highlight the importance of the piloting the CPR impact analysis prior to the start of the experimental phase.

### Phase IV: Dissemination/Long term implementation

If the impact analysis study shows a CPR to be effective then focus moves to the translation of the CPR from a research setting into everyday clinical practice delivered by the wider community of clinicians. Researchers should consider dissemination and implementation throughout the earlier phases. There is extensive published research on effective implementation strategies and these will not be covered in depth in this article [20]. One such strategy advocates an implementation pyramid with strategies such as CDSS at the very top of the pyramid as a way in which research can be implemented [21]. Inclusion of or reference to CPRs within guidelines may help dissemination on a local or national scale [6].

Re-evaluation of widespread implementation is integral to the success of the process. Once a rule is implemented its adoption may alter over time. Initially users may diligently fill in a checklist or score card before deciding on the appropriate course of action but this adherence may decrease as clinicians become more familiar with the content of the rule. An audit tool or continuous quality improvement programme can be used to measure uptake of the rule and can be useful to identify whether use of the rule is sustained. If not, it may be necessary to use reminders or refresher sessions to encourage longer term use. Use of such a tool would enable clinical outcomes to be measured and identify actual change in practice.

## Conclusion

Similar to the MRC Framework for Complex Interventions this four stage process aims to improve planning for and testing of the impact analysis and implementation of CPRs in clinical practice. It provides a structured approach to this topical and complex area of research. This framework is a step towards promoting CPR implementation in clinical practice and will continue to evolve as more impact analysis studies are undertaken and published.

## Competing interests

The authors declare that they have no competing interests.

## Authors' contributions

EW wrote the manuscript. SS had original idea for the framework. EW, SS, RP-S, CMG, PV, JV, ML participated in the draft outline and contributed to content. EW, SS, RP-S, CMG, PV, GC, JV, ML, TF corrected the manuscript. GC wrote Phase three. All authors read and approved the final manuscript

## Pre-publication history

The pre-publication history for this paper can be accessed here:

http://www.biomedcentral.com/1472-6947/11/62/prepub
